# Ubiquitous Colonic Ileal Metaplasia Consistent with the Diagnosis of Crohn’s Colitis among Indeterminate Colitis Cohorts

**DOI:** 10.18103/mra.v11i8.4188

**Published:** 2023-07-27

**Authors:** William A. Breaux, Maya A. Bragg, Amosy E. M'Koma

**Affiliations:** 1 Schools of Medicine, Meharry Medical College, Division of Biomedical Sciences, Nashville, Tennessee, Unite States of America.; 2 Department of Biochemistry, Cancer Biology, Neuroscience, and Pharmacology, Nashville, Tennessee, Unite States of America.; 3 Department of Surgery, Colon and Rectal Surgery, Vanderbilt University Medical Center, Nashville, Tennessee, Unite States of America.

**Keywords:** inflammatory bowel disease, indeterminate colitis, Ulcerative colitis, Crohn’s colitis, Human alpha-defensin 5

## Abstract

**Background:**

Inadequate differentiated diagnostic features of predominantly colonic inflammatory bowel diseases i.e., ulcerative colitis and Crohn’s colitis, may lead to inexact diagnosis of “indeterminate colitis”. About 15% of indeterminate colitis patients are diagnosed at colonoscopy, in colonic biopsies, and/or at colectomy. Managing outcomes of indeterminate colitis, given its unpredictable clinical presentation, depends on future diagnosis of colitis, Crohn’s colitis or ulcerative colitis.

**Objective:**

Overview the diagnostic efficacy of ectopic colonic ileal metaplasia and human α-defens 5 (DEFA5 alias HD5) for accurate delineation of indeterminate colitis into authentic Crohn’s colitis and/ or ulcerative colitis.

**Design:**

We describe a targeted protein for potentially differentiating indeterminate colitis into an accurate clinical subtype diagnosis of inflammatory bowel diseases i.e., ulcerative colitis and Crohn’s colitis.

**Patients:**

Twenty-one patients with the clinically inexact diagnosis of indeterminate colitis were followed, reassessed and data analyzed.

**Main outcome measures:**

We observed that (i) some patients had their original diagnosis changed from indeterminate colitis to either ulcerative colitis or Crohn’s colitis; and (ii) human α-defensin 5 is aberrantly overexpressed in Crohn’s colitis.

**Results:**

Fifteen of the twenty-one (71.4%) patients with indeterminate colitis had their inconclusive diagnosis changed; nine patients changed to ulcerative colitis and six to Crohn’s colitis. In human colon surgical samples, Human α-defensin-5 was significantly upregulated in Crohn’s colitis. In addition, Human α-defensin 5 processing enzyme, matrix metalloptotease-7 was inversely expressed compared to Human α- Defensin 5.

**Limitation:**

Due to the sequence homology of the α-defensin class of proteins, preceding efforts to raise antibodies (Abs) against DEFA5 have limitations to produce adequate specificity. The Abs used in previous assays recognizes the α-defensins, active α-defensins 5 and inactive pro- α-defensins 5. Monoclonal antibodies (mAbs) to determine specificity and sensitivity of α-defensins 5, which is diagnostic of CC disease, and NOT other α-defensins is the limitation to overcome.

**Conclusion:**

It is feasible to differentiate ulcerative colitis from Crohn’s colitis among patients with inexact diagnosis of indeterminate colitis using Human α-defensin 5 as a molecular biosignature delineator.

## Introduction

Inadequate differentiated diagnostic features of the two predominantly colonic inflammatory bowel diseases (IBD) i.e., ulcerative colitis (UC) and Crohn’s colitis (CC), may lead to an inexact/inconclusive diagnosis of indeterminate colitis (IC). 1,2 Managing phenotypic outcomes of IC, given its unpredictable clinical presentation and disease course, is challenging in endoscopic medicine. 1,2 A significant subgroup of IBD patients are misdiagnosed or are delayed to be diagnosed even when a combined state-of-the-art classification system of clinical, endoscopic, radiologic and histologic tools is used. 1,2 The objective of this overview is to discuss about the identified molecular-biomarkers that is likely to help clinicians more accurately differentiate the inexact IC diagnosis into authentic UC or CC. 3,4 In order to fulfill these objective, surgical human colon tissues was assayed for aberrant expression of proteins. These analyses provide quantitative and qualitative data about cellular systems, can potentially delineate diseases within the same organ during the patient's first biopsy visit to the clinic and may offer insight into potential causes of inflammation in CC. 3,4 Human α-defensin–5 (DEFA5 alias HD5) was the only gene to show up in both the microarray and the NanoString PCR array demonstrated that DEFA5 was increased 118-fold in CC versus UC, compared to 31-fold analyzed by microarray. 5

In colorectal surgery, the distinction between UC and CC is of utmost importance when determining a patient’s candidacy for pouch surgery, the restorative proctocolectomy with ileal pouch-anal anastomosis (RPC-IPAA). 6) Approximately 15-30% of patients whose UC or IC is surgically treated resolved with an ultimate diagnosis of CC, for which surgery may have been contraindicated. 7,8 Construction of RPC-IPAA in these patients often is painstaking due to multiple postoperative complications and may result in pouch failure range from 5% to 18% when the ultimate diagnosis is CC with significant morbidity. 7, 9,10 Change of diagnosis of IC to UC or CC is also observed in patients with IBD before colectomy, 11,12 as is the development of de *novo* Crohn’s ileitis after surgical intervention. 10 Incorrect diagnosis and treatment course carries potential morbidity from inappropriate and unnecessary surgeries, 8,13 and underscores the necessity of research efforts aimed at a more accurate diagnosis of the IBD. 3,4 This paper discusses a potential diagnostic molecular biomarker for delineating IC into an accurate clinical diagnosis of authentic UC or CC prior to appropriate care intervention. 3,4,14 These successful studies are the first of their kind to use DEFA5 in the colon microenvironment to delineate the IBD subtypes. The gene for DEFA5 was the single highest overexpressed gene in the CC profile.

DEFA5 is a human protein that is encoded by the *DEFA5* gene and is highly expressed in secretory granules of Paneth cells of the small intestine (ileum). 15 DEFA5 is a microbicidal and cytotoxic peptide involved in host defense mechanisms and is responsible for non-specific killing of microbes. 15,16

In determining levels of DEFA5 in the IBD, we overviewed its possible mechanism. It is known that MMP-7 is responsible for cleaving and activating DEFA5. 17,18,19 Although MMP-7 did not show up on the microarray, we sought to determine if levels of MMP-7 were also aberrant in IBD. Our data indicate that there is not a significant change in the levels of MMP-7 between UC and CC except when comparing moderate and severe CC to mild UC, but we observed an inverse relationship between levels of DEFA5 and Pro-MMP-7 in the IBD. The relationship between high levels of DEFA5 and low levels of MMP-7 in CC seems to suggest that there may be a dysfunction in the activation pathway of DEFA5 processing in CC, and therefore, gives us a potential mechanism for inflammation in CC.

To date, there is no available single molecular biomarker that has proven to have the desired diagnostic qualities in IBD. In this overview, we provide DEFA5 as a possible signature biomarker to solve the diagnostic ambiguity and delay in IBD. Several lines of evidence herein presented suggest that the use of *DEFA5*, also abbreviated as HD5, as a biomarker to molecularly differentiate CC from UC in otherwise inexact IC diagnosis is a conceptual innovative strategy for identifying appropriate patients with colonic IBD for recommended surgical treatment regimens.

## Methods

### Clinical Samples:

Studies were approved by the Meharry Medical College (IRB file number: 100916AM206) and Vanderbilt University (IRB file numbers: 080898 and 100581) Institutional Ethical Review Board Committees and conducted in accordance with the Second International Helsinki Declaration. 7,20 Informed consent in the initial studies was given, and participation was voluntary. The patients used in the studies were adults with definitive UC, CC and IC. 3,4 Full thickness surgical colectomy tissue samples were analyzed as previously described. 3,4

### Diagnostic criteria for inflammatory bowel disease

Pathology teams at Meharry Medical College (BRB, SDJ) and Vanderbilt University Schools of Medicine (MKW) used the following protocol criteria for the final surgical pathology reporting.

### Criteria for ulcerative colitis:

Characteristic pattern of involvement of colon, worse distally in untreated patients; lack of perianal or fistulizing disease; no granulomas, except in association with ruptured/injured crypts; no transmural lymphoid aggregates or other transmural inflammation; no involvement of terminal ileum, except mild “backwash ileitis” in cases with severe cecal involvement and no pyloric metaplasia in terminal ileum.

### Criteria for Crohn’s disease:

Involvement of other sites in GI tract (skip lesions, segmental disease); perianal or fistulizing disease; granulomas, not in association with ruptured/injured crypts and terminal ileum involvement.

### Criteria for indeterminate colitis:

Distribution favors UC, but focal transmural inflammation, or inflammation in ileum more than expected in backwash ileitis and no fistulizing disease.

### Vanderbilt Patient Medical Records Database:

The availability of a detailed IBD patient database registry at VUMC makes chart review and follow-up surveillance possible. Medical records data on patient demographics, preoperative variables prior to and after the time of RPC-IPAA surgery, surveillance endoscopic and clinical findings, and medical and surgical treatment history are able to be retrieved retrospectively. Further, VUMC has many health centers of practices located throughout greater Nashville metropolitan area and parts of Kentucky that provide referrals to the IBD center.

### Indeterminate colitis clinical retrospective study:

A retrospective investigation was conducted to identify a cohort of patients diagnosed with IC and registered in the IBD Center at VUMC. Twenty-one patients, initially classified as IC at the time of diagnosis between years 2000 - 2007, were identified and reevaluated for disease course in 2014 after a mean surveillance follow-up of 8.7±3.7 (range, 4-14) years in order to identify the rates of diagnosis resolution to UC or CC. Diagnosis for each patient was determined based on standard clinical and pathologic features as described in the diagnostic criteria in the methods section. 18,21 Three gastrointestinal pathologists blinded to clinical diagnosis reconciled and confirmed colitis diagnosis for each patient and represented a consensus among treating physicians. Patients who clinically did not change and maintained the IC diagnosis were molecularly tested for IBD phenotype precision.

### cDNA microarray:

Whole-transcriptome microarray with RNA extracted and pooled from human full thickness colon samples from UC and CC patients (n = 5/group) (Affymetrix, Santa Clara, CA) as previously described. 22,23

### nCounter gene expression:

_RNA from experimental and control cell was processed following NanoString (NanoString Technologies Inc., Seattle, WA) recommended procedure to determine gene expression level according to the manufacturer protocol. 20,24

### Real-Time RT-PCR:

qPCR was used to measure transcript levels of DEFA5 and MMP-7 for MicroRNA signatures to differentiate CC from UC as previously described. 5, 25,26,27 RNA was extracted from three human colon biopsy samples from moderate UC and CC and diverticulitis (DV) as a non-IBD control (RNeasy Miniprep Kit, Qiagen, CA). cDNA was generated using iScript cDNA synthesis kit (Bio-Rad, Hercules, CA). Predesigned TaqMan probes (Thermo Fisher Scientific, Waltham, MA) were purchased for HD5, MMP-7, and GAPHD control and all samples were run in triplicate using a CFX96 real-time PCR thermocycler (Bio-Rad). Data were analyzed according to the ΔΔC† method of analysis.

### Western blot and immunohistochemistry:

Western blot was utilized to assess any differences of DEFA5 and MMP-7 at the protein level as previously described (28) (29). Protein was extracted from a minimum of 10 colon biopsy samples from mild, moderate, and severe UC; mild, moderate, and severe CC; and non-IBD DV control. Whole cell lysates were extracted from full-thickness colon samples using T-PER (Thermo Fisher Scientific) according to manufacturer’s protocol. Bradford Assays (Bio-Rad) were run to determine protein concentration, and protein was loaded onto a 4-20% SDS-PAGE tris/glycine gel (Bio-Rad). Proteins were transferred to PVDF (Bio-Rad) and western blots for DEFA5, Pro-MMP-7, and β-actin loading control were performed with primary and secondary antibodies (Santa Cruz, Dallas, TX) according to manufacturer’s protocol. Blots were visualized with Opti-4CN colorimetric detection kit (Bio-Rad) and imaged with ChemiDoc XRS+ imaging system (Bio-Rad). Band intensities were measured and data analysis performed with Image Lab Software (Bio-Rad).

Five colon tissue protein extracts and staining of DEFA5 per disease by IHC was done as previously described. Quantification of DEFA5 staining was analyzed manually by microscopy / automatically quantified using Nikon's Eclipse Ti microscope with built-in NIS-Elements Advanced Research software (NEARAS). 22,28

### NEARAS Technology for Quantification of IHC Staining:

Nikon Elements Advanced Research Software (NEARAS) (Melville, NY) was used to manually calculate the number of cells with DEFA5 staining in IHC tissue slides. 22,28 A mean intensity threshold of 20 to 255 intensity units was established to eliminate a false positive signal from background staining. A circularity parameter of 0.5 to 1 and equivalent diameter of 5-15 micrometer was used to select for cells. All threshold parameters were used in each image to count the number of DEFA5 positive cells in tissue samples.

### Statistical analysis:

Statistical analyses were performed using GraphPad Prism v6 software (30) qRT-PCR, IHC DEFA5 counts, and DEFA5 vs. MMP-7 protein analysis was examined by applying a two-tailed Student’s t test with the Welch correction, respectively (qRT-PCR and IHC = unpaired; DEFA5 vs. MMP-7 analysis = paired). Western blots were analyzed by ANOVA followed by Fisher’s test for multiple comparisons. For all statistical analyses, p< 0.05 indicated a statistical significance.

## Results

**Fig 1:**
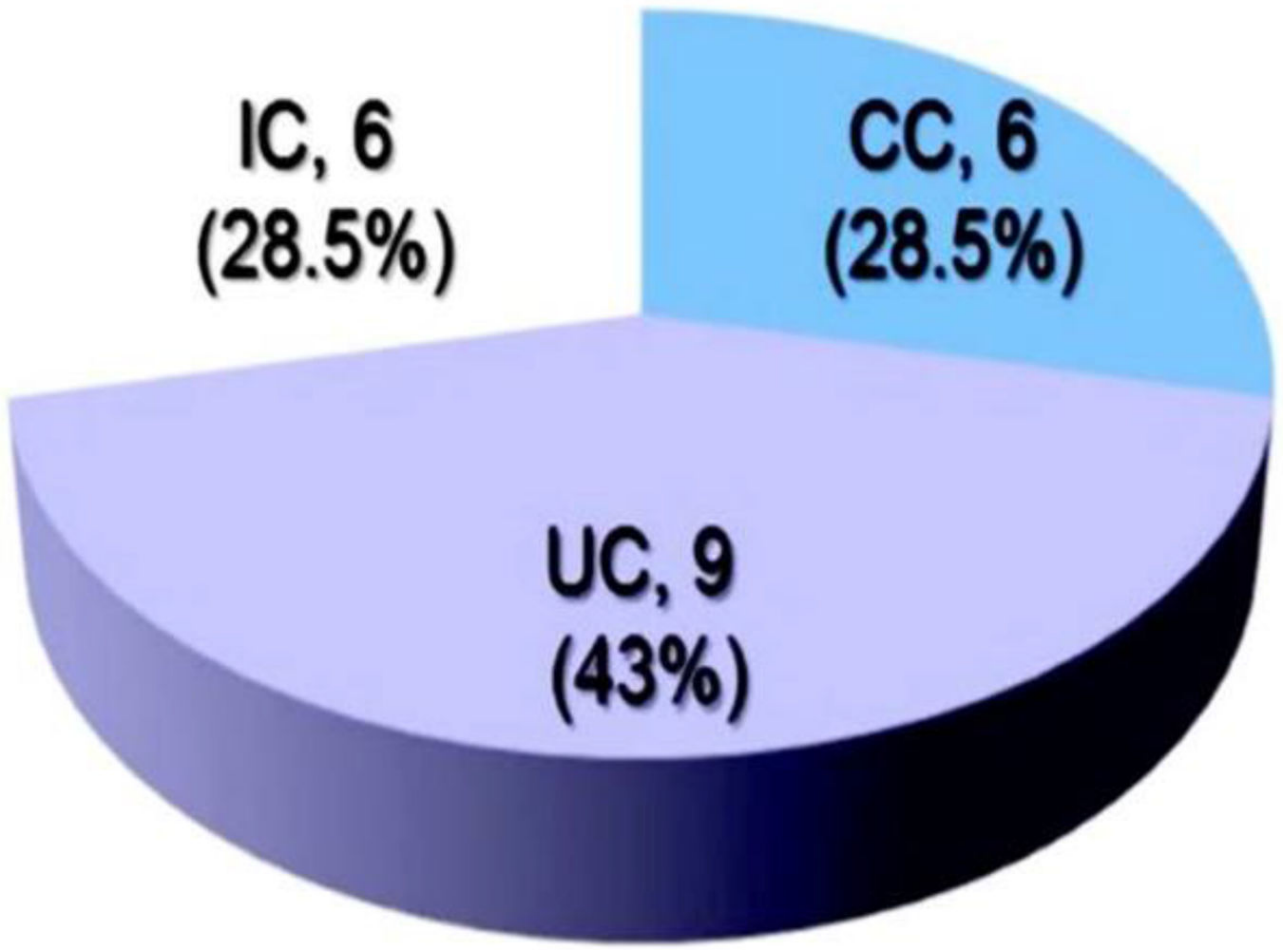
Depict diagnostic ambiguity, uncertainty and inaccuracy of IC in IBD clinical setting. **A**, Twenty-one IC patients were followed for approximately ten years. At the end of the 10 year period, 28.5% of the patients could still not be delineated into a precise diagnosis of either UC or C. Immunohistochemistry staining for DEFA5 agrees with final diagnostic outcome in a sample of IC patients even when there was no agreement with the attending physician. Adapted from Williams *et al. 5* under the terms of conditions of the Creative Commons Attribution license.

**Fig 2:**
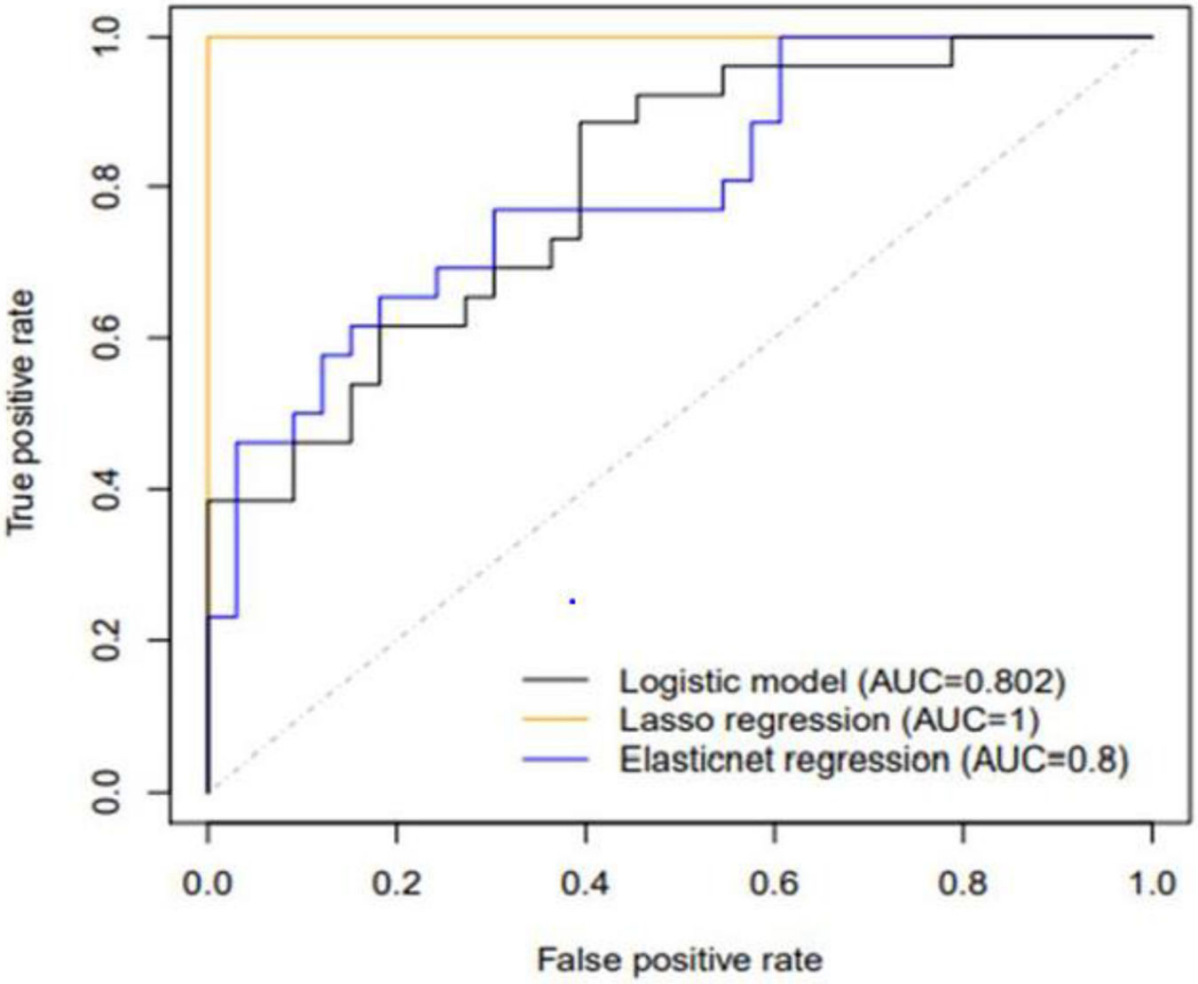
Statistical analysis to determine positive predictive values (PPVs) of DEF5 in patient tissue are 95.8% for CC and only 76.9% for UC. Chi squared analysis shows significant relatedness between high levels of DEFA5 and a diagnosis of CC (*p<0.0001*). These data indicate that DEFA5 could be developed into a diagnostic tool to better distinguish CC *from UC*. This indicates that IC could be circumvented and eradicated for good. Adapted from Williams *et al. 5* under the terms of conditions of the Creative Commons Attribution license.

Twenty-one (#21) patients, Table 1 who were diagnosed with IC were reevaluated after a surveillance follow-up period was 8.7±3.7 (range, 4-14) years. Seventy-one percent (#15) of the patient with the original IC diagnosis changed. Forty three percent (# 9) changed to UC and another 28.5% (# 6) to CC, respectively, Table 1 and Fig. 1. Twenty nine percent (# 9) patients remained with the inexact diagnosis of IC, Table 1 and Fig. 1. When the initial biopsies obtained from the 6 unchanged patients were analyzed for DEFA5 IHC NEARAS count profile tests, all reconciled with the final diagnosis chart reviewed in the Database. Among these patients with IC, statistical analysis of DEFA5 was a reliable differentiator to determine positive predictive values (PPV) in patient tissue was 95.8% for CC and only 76.9% for UC, Fig. 2. These data indicate that DEFA5 could be developed into a reliable diagnostic tool to better distinguish CC and UC among IC patient cohort.

## Discussion

Diagnostic ambiguity and delay among IBD patients with IC has remained a major challenge in endoscopic medicine 31,32,33,34,35 and colorectal surgery. 36,37,38 The central medical challenge is the discrimination of inexact IC diagnosis into the specific IBD subtypes (UC or CC) with accuracy, as it affects surgical care of patients. 36,37,38 Experiences reveals 28.5% of patients with inexact IC diagnosis still retained their IC diagnosis after a mean follow-up period of 8.7±3.7 years (range: 4-14), Fig. 2B (5) (39). Diagnosis of IC into accurate diagnosis of authentic UC or CC is of paramount importance when determining a patient’s candidacy for pouch surgery, the RPC-IPAA. Further, incorrect diagnosis and treatment carry potential morbidity from inappropriate and unnecessary surgery and cost. The overall colonic IBD diagnosed as IC has not changed over the past 60 years, revealing persistent diagnostic uncertainty, thereby confounding effective therapies. 5, 27

A standard surgical procedure for treating UC, RPC-IPAA typically does not benefit and can be harmful to most CC patients because of higher rates of pouch failure. Therefore, it is important for surgeons to choose the appropriate treatment for their IBD patients 40, 41, especially IC into authentic UC or CC. Unfortunately, current methods for diagnosing colonic IBD are painstakingly inaccurate and have not changed over 60 years. 42,43 Up to 15% of IBD cases are classified as IC when the established criteria for UC and CC are non-definitive. 5,43 In another 15% of IBD cases, authentic CC is not evident prior to colectomy. 1, 44 Therefore, a total of 30% of colonic IBD patients are not accurately diagnosed in a timely manner. 1,44 Much of the diagnostic uncertainty arises from overlapping features that make CC appear like UC. 45,46 Key differences in tissue inflammation, damage, and prognosis, which suggest distinctive etiopathogenic processes and mechanisms responsible for their respective features, can clinically and histologically be challenging to interpret. 43,47 Intestinal wall thickening is segmental in CC but continuous in UC. 45,48 UC causes inflammation and ulceration of the mucosal and, to a lesser degree, the submucosae linings of the colon and rectum. 45 Furthermore, CC differs from UC in that it may cause inflammation deeper within the four colonic layers (transmural inflammation and skip lesions). 49,50 CC may also affect other organs through fistulation. 49,50 Unfortunately, during the prodromal stages of the disease, all these features are obscure in 30% of cases confounding the treatment regimens. 45,46 The surgical treatment options and indications for UC and CC differ significantly; for instance, UC patients need to undergo RPC-IPAA for cure which largely is contraindicated in patients with CC. 6,51,52 Therefore, it is important to accurately identify the IBD subtypes UC or CC and categorize inexact diagnosis of IC into authentic either UC or CC when determining a patient’s candidacy for RPC-IPAA surgery.

To circumvent the diagnosis ambiguity and delay in IBD clinical setting we analyzed colonic surgical pathology samples of patients with unambiguous CC and UC undergoing colectomy in connection with RPC. 3,4 We identified and compared those protein profiles which had the necessary (i) specificity; (ii) sensitivity; (iii) discriminatory; and (iv) predictive capacity to determine the heterogeneity of IBD. 3,4 These molecules are independent of tissue (mucosa, submucosa, or both) and appear to represent a disease-specific marker. 3,4. Further, in this study, we were able to molecularly delineate UC and CC with molecular signatures of DEFA5 using IHC and quantified by NEARAS.

To date, there is no diagnostic “Gold standard” tool for IBD. Differentiating UC and CC among patients with IC has remained a major challenge in endoscopic medicine. 53,54 Patients with CC are mistakenly diagnosed and RPC-operated as definitive UC in 15% of IBD patients because of overlap in the clinical findings. 13,55,56,57 Further, most IC patients who undergo RPC for presumed UC, are subsequently found to develop a recurrent de novo Crohn’s ileitis in the ileal pouch. 13,58 This is a serious consequence that may hinder the restoration of intestinal continuity and its intractable nature leads to subsequent pouch failure, 7,8,59 often requiring pouch diversion or excision with a permanent terminal-ileostomy. 13,56,60,61,62,63,64,65 This has negative psycho-sociological implications and poorer quality of life. 60,66

Curative treatment for UC is often surgical. 67 Success of RPC surgery is largely dependent on careful patient selection combined with meticulous surgical technique and diagnostic accuracy. 68 Available clinical presentations and experience to date suggest that it is difficult to identify patients with CC who are likely to have a successful outcome after RPC surgery. 13,56 However, in a highly selected patient with CC, RPC has been indicated. 69 Our biomolecular marker model may ultimately be used to select those patients with CC who are potential candidate for this sphincter preserving operation, RPC. Thus, RPC operation may be considered and should remain a careful option for certain subgroup of patients with CC, 69 but an acceptable care option for patients with UC and for those IC patients who are predicted to develop UC. 6,70

UC and CC share many demographic and clinical features yet present significant differences in tissue inflammation and damage, suggesting a distinct etiopathogenic trigger. 71 Currently, little is known about the molecular differences distinguishing UC and CC. 3,4 Trends in the IBD field focus on genetic susceptibility, role of normal flora, inflammatory processes, and interactions between normal flora and the immune response. 72 Even though current research is promising, there have been no definitive answers to date to help clinicians differentiate between the two diseases when current diagnostics prove inadequate and result in a diagnosis of IC. As incidence and prevalence of IBD increases across the world, it is becoming even more important to find molecular markers of disease to accurately distinguish between CC and UC. 3,4 Our studies led to the discovery of DEFA5 as a potential biomarker for CC and that may be a diagnostic signature that may efficiently distinguish CC from UC as “Gold Standard” tool in IBD phenotype precision. In addition to a novel diagnostic indicator for IBDs, we also propose a dysregulation in the activation of DEFA5 in IBD. Due to the low levels of MMP-7 in moderate and severe CC, it is possible that DEFA5 is remaining localized in the tissue in its inactive form, which could be cause for an increase in damage to the epithelial lining and potentially even a dysregulation in the levels and make-up of gut flora. Future studies will also focus on discovering if there is a causative relationship of these two proteins.

Contrary, in line with the discussed data herewith presented, it is evidently observed in a newly assembled cohort that patients with “Crohn’s ileitis (CI)” are characterized with a deficiency of DEFA5, (73) as shown by a reduced expression and secretion of the Paneth cell defensing DEFA and, is a fundamental feature of CI. In Crohn’s colitis (CC), mutually opposed, the reverse is true. We found DEFA5 to be the most predominantly expressed antimicrobial peptide. Lawrance at al. 14 had similar observation when they examined global gene expression profiles of inflamed colonic tissue using DNA microarrays. The results identified several genes with altered expression between UC and CD. DEFA5 gene was the most highly expressed in CD. Unfortunately, MMP-7 was not mentioned but MMP-1, 3 and 12 transcripts were significantly higher in UC than the control (*p* < 0.05). The definitive CC and CI diseases do not share demographic and clinical features and/or molecular biometrics of tissue inflammation and damage suggesting distinct etiology and mechanisms. This is intriguing and needs further elucidation. These studies have reconciled all IC patient samples into authentic either UC or CC using DEFA5 and patient outcomes, Table 1 and Fig. 1.

Early timely diagnosis accuracy leads to appropriate therapeutic options and will aid in determining candidates for RPC-IPAA and avoid unnecessary surgeries. 13,56 We have identified that DEFA5 can differentiate CC and UC amomg IC patients with the first clinic visit endoscopy biopsy without ambiguity or delay. Overall, significant differences in expression profiles of 546 genes identified CC and UC as distinct molecular entities and as yet unexplored pathobiologies and IBD-predisposing candidate genes. 5

## Conclusion

DEFA5 expressed differentially in IBD and that aberrant expression, localization, and/or activation of DEFA5 underlies the tissue inflammation and damage associated with authentic CC form of colitis. DEFA5 is a reliable signature to distinguishCC from UC among IC patient cohort. It is promosing to use DEFA5 to determine patient candidacy for RPC-IPAA surgery.

## Limitation

The high degree of similarity of DEFAs implies that antibodies against DEFA5 may not be specific enough to distinguish DEFA5 from other DEFAs. The rate limiting step is the generation of monoclonal antibodies to the DEFA5 peptides. We are confident that antibodies against the segments of DEFA5, dones 1A8 and 4FD will be successful. 74,75, 76 We have verified that the antibodies used to generate the data (DEFA5 – Sigma HPA015775) were indeed directed against DEFA5.

## Figures and Tables

**Table 1:** Results from patients with the diagnosis of indeterminate colitis samples that were surveyed: Column (i) original diagnosis by a pathologist, (ii) diagnosis by an attending physician (iii) diagnosis using molecular biomarker test, DEFA5 staining (each + represents 100 staining spot counts), and (iv) patient clinical outcomes. Adapted with modification from Williams *et al., 5* under the terms of conditions of the Creative Commons Attribution license.

Attending Pathologist Diagnosis	Attending Gastroenterologist Diagnosis	Patient Outcomes New Diagnosis	Mean Area Fraction of DEFA5 (%) NEARES count
			
IC	IC	CC	80
IC	UC	UC	10
IC	UC	UC	10 (Second opinion)
IC	UC	UC	20
IC	UC	UC	20 (Second opinion)
IC	IC	UC	20
IC	UC	UC	20
IC	CC	UC	10
IC	CC	UC	10
IC	UC	CC	70 (Second opinion)
IC	UC	CC	70
IC	UC	UC	20
IC	CC	CC	90
IC	IC	CC	80
IC	CC	CC	80
IC	UC	UC	10
IC	CC	CC	100
IC	UC	UC	20
IC	CC	CC	100
IC	IC	UC	20
IC	IC	CC	100
IC	IC	UC	10
IC	UC	UC	20

## Abbreviations

IBD: inflammatory bowel disease
IC: Indeterminate colitis
UC: Ulcerative colitis
CC: Crohn’s colitis
CD: Crohn's disease
CI: Crohn’s ileitis
DV: Diverticulitis
NEARAS: Nikon Element Advance Research Analysis Software
RPC: restorative proctocolectomy
IPAA: ileal pouch-anal anastomosis
H & E: hematoxylin and eosin
HD5: Human Defensin 5α
ROC: receiver operator characteristic
MMC: Meharry Medical College
VU: Vanderbilt University
VUMC: Vanderbilt University Medical Center
